# The Annual of the Universal Medical Sciences for the Year 1889

**Published:** 1890-02

**Authors:** 


The Annual of the Universal Medical Sciences for the year 1889 are just at hand. This issue is a very great improvement over that of 1888, and to-day it is without doubt the best resume of the year's work in the medical sciences published in this or any other language. Every branch and subject is treated, and with the many cuts illustrating the text it becomes one of the most useful of books to the busy practitioner. F. O. S.

